# *Bacteroides intestinalis* DSM 17393, a member of the human colonic microbiome, upregulates multiple endoxylanases during growth on xylan

**DOI:** 10.1038/srep34360

**Published:** 2016-09-29

**Authors:** Kui Wang, Gabriel V. Pereira, Janaina J. V. Cavalcante, Meiling Zhang, Roderick Mackie, Isaac Cann

**Affiliations:** 1 Energy Biosciences Institute, University of Illinois at Urbana-Champaign, Urbana, IL 61801, USA; 2 Carl R. Woese Institute for Genomic Biology, University of Illinois at Urbana-Champaign, Urbana, IL 61801, USA; 3 Department of Animal Science, University of Illinois at Urbana-Champaign, Urbana, IL 61801, USA; 4 Department of Microbiology, University of Illinois at Urbana-Champaign, Urbana, IL 61801, USA

## Abstract

Many human diets contain arabinoxylan, and the ease of genome sequencing coupled with reduced cost have led to unraveling the arsenal of genes utilized by the colonic Bacteroidetes to depolymerize this polysaccharide. The colonic Bacteroidetes with potential to ferment arabinoxylans include *Bacteroides intestinalis*. In this study, we analyzed the hydrolytic activities of members of a xylan degradation cluster encoded on the genome of *Bacteroides intestinalis* DSM 17393. Here, it is demonstrated that a cocktail of the xylanolytic enzymes completely hydrolyze arabinoxylans found in human diets. We show that this bacterium and relatives have evolved and secrete a unique bifunctional endoxylanase/arabinofuranosidase in the same polypeptide. The bifunctional enzyme and other secreted enzymes attack the polysaccharides extracellularly to remove the side-chains, exposing the xylan backbone for cleavage to xylo-oligosaccharides and xylose. These end products are transported into the cell where a β-xylosidase cleaves the oligosaccharides to fermentable sugars. While our experiments focused on *B. intestinalis*, it is likely that the extracellular enzymes also release nutrients to members of the colonic microbial community that practice cross-feeding. The presence of the genes characterized in this study in other colonic Bacteroidetes suggests a conserved strategy for energy acquisition from arabinoxylan, a component of human diets.

The human lower gastrointestinal tract (GIT) harbors trillions of bacteria that orchestrate a wide variety of biological activities absent in the host human cells^1^,^2^,^3^,^4^,^5^. Moreover, humans lack enzymes required to degrade and ferment structural polysaccharides present in dietary fibers^6^. Fermentation of dietary fibers by the microbes residing in the human GIT represents approximately 3–10% of the human energy balance^7^, and has a large impact on the microbiota diversity in the GIT. It has been reported that the GIT microbiota have influence over development of the human immune system, physiology and metabolism^8^. An unbalanced microbiota, usually termed dysbiosis, has been linked to chronic GIT diseases^9^, allergies^10^, obesity^11^, and colorectal cancer^12^. Recent advances in DNA sequencing has unraveled the complex microbial communities in the GIT, leading to microbial gene libraries that surpass the genes encoded in the human genome by 100-fold^4^,^13^. It is estimated that roughly 75% of the genes encoded by the human gut microbiome are of unknown function or are poorly characterized^4^.

Fermentation of dietary fiber is one of the most important roles of the human lower GIT microbiota because they can degrade polysaccharides that are recalcitrant to human enzymes^14^. Xylan is a hemicellulose, the second largest structural polysaccharide on our planet, and it is found in especially significant amounts in cereal grains consumed by humans, such as wheat, rye, and oat^15^. Due to its heterogeneity, complete hydrolysis of xylan to its unit sugars requires a combination of different enzymes such as endoxylanases, β-xylosidases, ferulic acid esterases, α-glucuronidases, α-L-arabinofuranosidases, and acetylxylan esterases^16^. A total of roughly 168 different catalytic modules or polypeptides related to the degradation of plant structural polysaccharides, including xylan, have been described across all three domains of life, and the vast majority is found in microbial communities^17^. The enzymatic activities in these polypeptides include glycoside hydrolases (GH), polysaccharide lyases (PL), glycoside transferases (GT), and carbohydrate esterases (CE), and these polypeptides are frequently associated with one or more of 64 families of carbohydrate binding modules^17^,^18^.

In the human lower gut microbial community, the genus *Bacteroides* encodes the largest collection of genes related to xylan degradation^18^,^19^. These organisms have a large variety of polysaccharide utilization loci (PULs) composed of core genes encoding carbohydrate/polysaccharide binding proteins, extracellular polysaccharide degrading enzymes, outer membrane transporters, hybrid two component systems (HTCS) containing polysaccharide sensing modules, and a repertoire of glycoside hydrolases in the periplasm that breakdown oligomeric sugars into fermentable sugars^20^,^21^. This conserved xylan utilization system (XUS) is present in several members of the genus *Bacteroides* in the GIT of a range of hosts^14^,^19^,^22^.

*Bacteroides intestinalis* DSM 17393 is a xylanolytic bacterium isolated from the fecal sample of a healthy Japanese through a selective medium for polyamine-producing bacteria^23^. This bacterium possesses a large genomic region (~60 kb) shown to target xylan degradation, and in addition it is thought to encode the largest aggregation of carbohydrate-active enzymes (CAZymes) among all gut bacteria to date^14^,^24^. Previous work, including transcriptome analysis of *B. intestinalis* DSM 17393 grown on wheat arabinoxylan (WAX), demonstrated that nearly 70 genes related to xylan degradation and spanning two large distinct PULs, were up-regulated when compared to the same organism grown on xylose. In the present study, we present the function and comparison of five different endoxylanases (BACINT_04197, BACINT_04202, BACINT_04210, BACINT_04213, and BACINT_04215) upregulated in one of the two PULs during growth on xylan versus xylose. Furthermore, we determine the functional interactions of the endoxylanases with two putative accessory enzymes (BACINT_04203 and BACINT_04205) encoded in the same PUL and upregulated with the endoxylanases in *B. intestinalis* DSM 17393. By comparing the domain architectures of the endoxylanases, assessing their biochemical activities, and elucidating their synergistic interactions with the putative accessory enzymes, we provide insights into the enzymatic machinery utilized by a human gut commensal bacterium in depolymerizing polysaccharides found in human diets. Energy acquisition and cellular building blocks from the metabolism of the depolymerized polysaccharides form an important prerequisite for establishment and persistence of the human lower gut microbiome.

## Results

### Multiple endoxylanases in a gene cluster in the human commensal bacterium *B. intestinalis* DSM 17393

**Figure 1:**
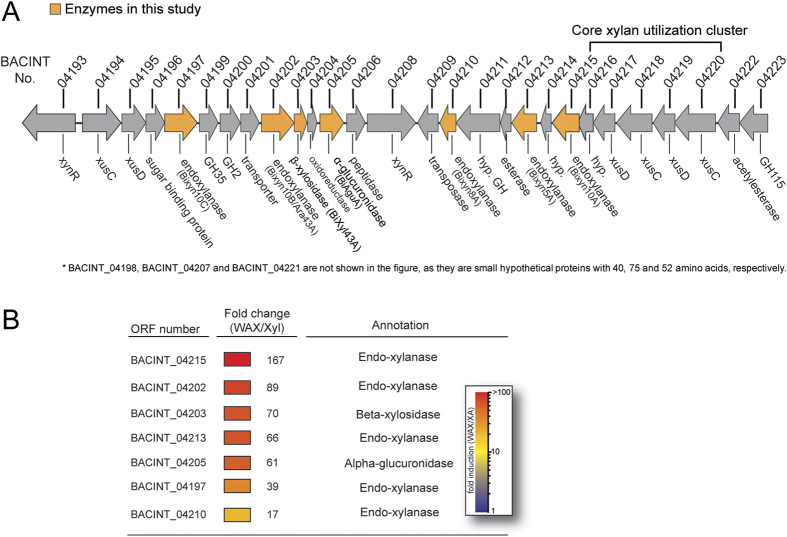
Five xylanases, β-xylosidase and α-glucuronidase encoding genes in *B. intestinalis*. (**A**) The genomic context for the xylanolytic enzyme coding genes in *B. intestinalis*. (**B**) Fold change (up-regulated) of the xylanases, β-xylosidase and α-glucuronidase genes transcription on soluble WAX compared with xylose.

The major human gut bacterium *B. intestinalis* DSM 17393 has been shown to up-regulate a large number of genes during growth on xylan^14^. Among the up-regulated genes is a group that clusters on the genome (Fig. 1A). Within the cluster are several genes that are predicted to encode endoxylanases and their accessory enzymes for degradation of xylans. The accessory enzymes are putative side-chain removing enzymes, i.e., a predicted β-xylosidase or an arabinofuranosidase and a putative α-glucuronidase. The xylan targeting genes and their predicted functions are BACINT_04215 (endoxylanase), BACINT_04202 (endoxylanase), BACINT_04203 (β-xylosidase), BACINT_04213 (endoxylanase), BACINT_04205 (α-glucuronidase), BACINT_04197 (endoxylanase), and BACINT_04210 (endoxylanase), and they were up-regulated 167-, 89-, 70-, 66-, 61-, 39-, and 17-fold during growth on wheat arabinoxylan (WAX) relative to xylose (Fig. 1B). Since endoxylanases are critical to the harvesting of the energy in xylans, nutrients inaccessible to the human host cells, we explored how *B. intestinalis* endoxylanases act in concert with the co-clustering accessory enzymes to degrade xylans present in three cereals consumed by humans as energy source.

### Modular organization of the *B. intestinalis* DSM 17393 putative endoxylanases

**Figure 2:**
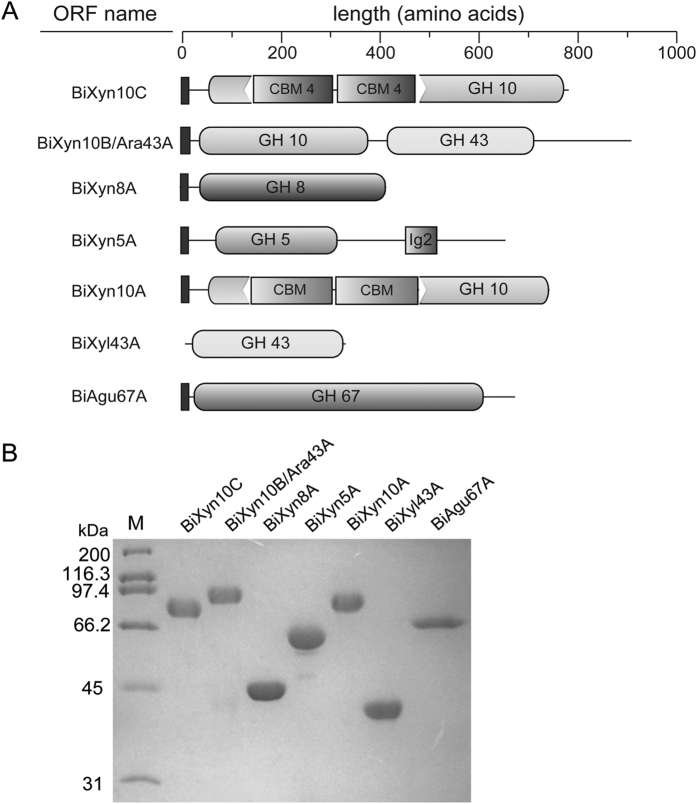
Xylanolytic enzymes of *B. intestinalis*. (**A**) Schematic domains of five putative endoxylanases (BiXyn10A, BiXyn5A, BiXyn8A, BiXyn10B/Ara43A, and BiXyn10C) and two accessory enzymes (BiXynl43A and BiAgu67A) (**B**) SDS-PAGE analysis of the seven xylanolytic enzymes mentioned above in panel A.

In Fig. 2A, we present the modular organization of the seven polypeptides in the cluster that likely targets xylans for degradation. The gene product of BACINT_04197 or BiXyn10C is made up of a predicted signal peptide and a glycoside hydrolase family 10 (GH10) module with an insertion of two family 4 carbohydrate binding module (CBM)-like sequences. The BiXyn10B/Ara43 polypeptide is made up of a putative N-terminal signal peptide followed by a GH10 module and a GH43 module (annotated as a β-xylosidase in the Genbank). The BiXyn8A is composed of a putative N-terminal signal peptide and a GH8 module. This protein was previously characterized together with another GH8 found in the same bacterium by our group^25^. The BiXyn5A comprises of a putative N-terminal signal peptide, a GH5 module and then an Ig2-like module. The final endoxylanase is very similar to BiXyn10C, as it is made up of a putative signal peptide and a GH10 module interrupted by two CBM sequences. The functions of this polypeptide including its inserted CBMs have been well characterized by our lab^14^. While BiXyl43A is composed of a GH43 module without a signal peptide, the other potential accessory enzyme BiAgu67A contains a signal peptide followed by a GH67 module. Thus, based on the modular organizations, except for Xyl43A, it is likely that each of the xylan targeting polypeptides characterized in the present study, is secreted outside of the cytoplasm. The Xyl43A on the other hand likely has a role inside the cytoplasm, as it lacks a signal peptide. Each protein was expressed with an N-terminal 6His-tag to facilitate purification by immobilized metal affinity chromatography (IMAC). The polypeptides were further purified by size exclusion chromatography close to homogeneity (Fig. 2B), as judged by sodium dodecyl polyacrylamide gel electrophoresis (SDS-PAGE).

### Optimum pH and temperatures of the xylan degrading enzymes in *B. intestinalis* DSM 17393

The optimum pH of the endoxylanases were determined at 37 °C with WAX as the substrate. In general, of the five endoxylanases, four exhibited a broad optimum pH range (Supplementary Fig. 1A). Thus, over 75% of the activity for Xyn10C, Xyn8A, Xyn5A and Xyn10A remained between pH 5.0 and 7.0, with the optimum pH occurring at 7.0, 6.0, 6.0, and 6.0, respectively, for the four enzymes (Table 1). The Xyn10B/Ara43A, on the other hand, exhibited a sharp pH optimum at 5.5, although over 85% of the enzymatic activity was detectable at pH 6.0. In the case of Xyl43A, the pH optimum was determined with xylobiose at 37 °C. With this substrate, Xyl43A showed an optimum pH of 6.5 (Table 1), with over 65% of the maximum activity being detected at a pH range of 5.5–8.0. (Supplementary Fig. 1A).

Except for Xyl43A which showed an optimum temperature at about 35 °C, Xyn10C, Xyn10A/Ara43A, Xyn8A, Xyn5A, and Xyn10A showed optimum temperatures around 45–50 °C (Supplementary Fig. 1B). At 40 °C, we detected about 65%, 85%, 90%, 60%, 60%, and 80% of the maximum activity of Xyn10C, Xyn10B/Ara43A, Xyl43A, Xyn8A, Xyn5A, and Xyn10A, respectively (Table 1).

### Specific activities of multiple endoxylanases on xylan substrates

Analyses of the hydrolytic activities of the five enzymes were performed by determining the specific activity of each enzyme in its optimal pH and temperature. As shown in Table 2, all five enzymes exhibited hydrolytic activity with WAX, rye arabinoxylan (RAX), and oat spelt xylan (OSX). The Xyn10B/Ara43A and Xyn8A showed high specific activities towards all three substrates, and the highest activities were found with WAX. While Xyn10C, Xyn5A, and Xyn10A exhibited similar substrate specificities, they were more efficient in the hydrolysis of RAX than WAX. Furthermore, the specific activity with OSX, in each case, was less than that with WAX and RAX, suggesting that OSX is more recalcitrant to hydrolysis by the enzymes.

### Hydrolysis of xylans by the endoxylanases of *B. intestinalis* DSM 17393

**Figure 3:**
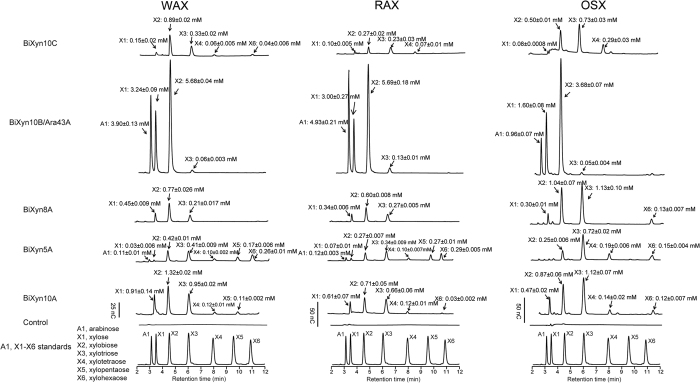
Overnight hydrolysis (14 hours) of xylan substrates (WAX, RAX and OSX) by the five putative endoxylanases. The experiments were carried out by incubating 5 mg/ml xylan substrate with each endoxylanase at final concentration of 0.5 μM at 37 °C for 14 hours, the products of hydrolysis were identified and quantified by HPLC, Arabinose (A1), xylose (X1) and xylo-oligosaccharides (X2 to X6) were mixed and analyzed by HPLC to serve as standards for the assignment of the released products. Calibration curves were constructed with known concentrations of A1 and X1-X6. The concentrations of each sugar were calculated by fitting the peak area into calibration curve.

We examined the hydrolytic patterns of the five endoxylanases at pH 6.5 and a temperature of 37 °C on the three different xylan sources derived from cereal grains, i.e., WAX, RAX, and OSX. The pattern of end products release from the three xylans by Xyn10C, Xyn5A, and Xyn10A were similar, with Xyn10A releasing more end products than the Xyn10C and Xyn5A enzymes (Fig. 3). The end products of the Xyn5A, on the other hand, consistently contained a product similar to xylopentaose, which was often missing in the end products of the Xyn10 enzymes. The Xyn8A released end products with retentions times similar to xylose, xylobiose and xylotriose; although on OSX, an end product similar to xylohexaose was also observed. The hydrolytic activity of BiXyn10A/Ara43A was quite different from the other xylanases, since it released large amounts of xylose and xylobiose from the three different substrates together with large amounts of arabinose, an end product not seen with the other endoxylanases. Note that discernible amounts of xylotriose were also released from all three substrates by BiXyn10A/Ara43A (Fig. 3).

### Hydrolytic products of the accessory enzymes clustering with the endoxylanases

**Figure 4:**
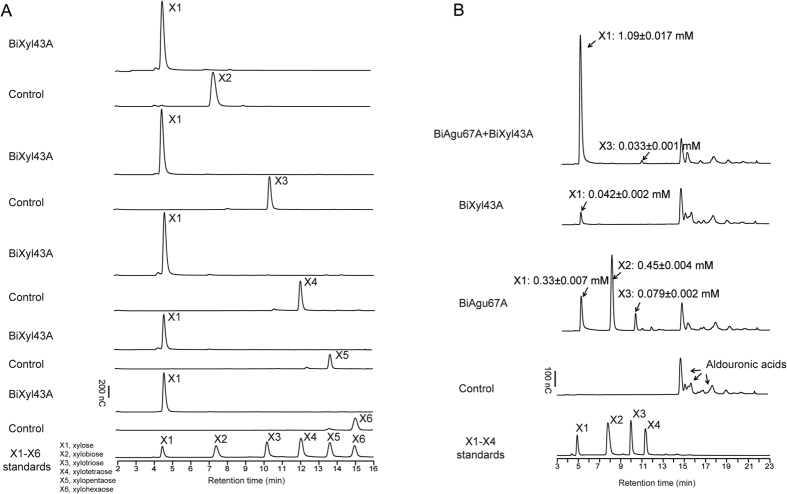
Functional analyses of *B. intestinalis* BiXyl43A and BiAgu67A. (**A**) Hydrolysis of xylo-oligosaccharides (X2–X6) by BiXyl43A. Purified BiXyl43A was incubated with different xylo-oligosaccharides and the hydrolytic products were analyzed by HPLC. (**B**) Hydrolysis of aldouronic acids by BiXyl43A and BiAgu67A. The activity of the BiAgu67A was determined by the function to release xylo-oligosaccharides from aldouronic acids and its capacity to release xylose from aldouronic acids when act synergistically together with a β-xylosidase (BiXyl43A). The reaction products were analyzed by HPLC and identified by compared with peaks with retention times of xylo-oligosaccharides as standards.

The gene products of the two genes predicted to encode accessory proteins in the cluster were examined for their functional roles in the degradation of the polysaccharide xylan, by using alduronic acids and also xylo-oligosaccharides ranging from xylobiose to xylohexaose. The alduronic acids were composed of short xylo-oligosaccharides with methyl-glucuronic acid side chains. As shown in Fig. 4A, the incubation of Xyl43A with xylo-oligosaccharides ranging from xylobiose to xylohexaose led to cleavage or hydrolysis to their unit sugar, i.e., xylose. The results, therefore, identified Xyl43A as a versatile β-xylosidase rather than an arabinofuranosidase. The Agu67A, predicted to be an α-glucuronidase, was tested for activity through its capacity to cleave the methyl-glucuronic acid side chains from the alduronic acids. As shown in Fig. 4B, incubating Agu67A with the alduronic acids resulted in the release of xylose and xylo-oligosaccharides (xylobiose and xylotriose) into the reaction mixture. In contrast, addition of Xyl43A to the alduronic acid led to release of only minor amount of xylose into the medium, indicating that the Xyl43A has the capacity to cleave xylose from some of the alduronic acids, most likely terminal xylose free of side chains. The results, therefore, demonstrated that Xyl43A does not cleave xylo-oligosaccharides decorated with methyl-glucuronic acids, most likely due to steric hindrance of the side chain in the active site. The two enzymes (Xyl43A and Agu67A), however, functioned synergistically to cleave the alduronic acids to mostly xylose (Fig. 4B).

### Synergistic activity of the xylan targeting enzymes clustering on the *B. intestinalis* DSM 17393 genome

**Figure 5:**
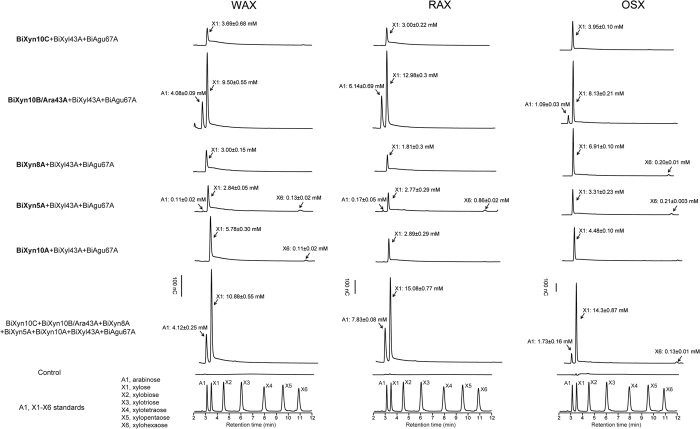
Overnight synergistic hydrolysis (14 hours) of xylan substrates of multiple endoxylanases with BiXyl43A and BiAgu67A towards WAX, RAX and OSX. The synergistic effect of the xylanases with the two putative accessory enzymes (BiXyl43A and BiAgu67A) were investigated by incubating BiXyl43A and BiAgu67A with each endoxylanase together at 37 °C for 14 hours, the synergistic hydrolysis experiments of all seven enzymes were performed as well. The final concentration for each enzyme was 0.5 μM, the final hydrolysis products were identified and quantified by HPLC, Arabinose (A1), xylose (X1) and xylo-oligosaccharides (X2 to X6) were mixed and analyzed by HPLC to serve as standards for the assignment of the released products. Calibration curves were constructed with known concentrations of A1 and X1–X6. The concentrations of each sugar were calculated by fitting the peak area into calibration curve.

We examined the functional interactions of the five endoxylanases and their associated accessory enzymes located in the gene cluster. In each experiment, the enzyme mixtures were incubated with the substrates at 37 °C for 14 hours. Combining each of Xyn10C, Xyn8A, Xyn5A or Xyn10A with the two accessory enzymes (Agu67A and Xyl43A) led to the release of xylose from each xylan substrate (Fig. 5). Combining the Xyn10B/Ara43A with the accessory enzymes, on the other hand, led to two- or more fold of the xylose released compared with the other mixtures. In addition, large amounts of arabinose were also released from each substrate in the presence of the bifunctional enzyme Xyn10B/Ara43A. A combination of the five endoxylanases with the accessory enzymes yielded a similar end products profile of xylose and arabinose from the three xylans, and the levels of the end products released were higher than any of the three enzyme-combinations. The accumulation of xylose was higher in RAX and OSX than in WAX. However, more arabinose was released from WAX and RAX compared to OSX. The differences in xylose and arabinose levels in the end products are likely due to the differences in the composition of the two sugars in the three different substrates, with WAX and RAX containing more arabinose than OSX^26^.

The forgoing results were determined for end point assays (14 hours incubations); therefore, we examined the end products from shorter incubations, i.e. 30 mins, to gain insights into the hydrolytic steps of the various enzymes. The hydrolytic pattern of Xyn10C at 30 mins was not different from that of the incubation for 14 hours, although more end products were present in the longer incubation (Supplementary Fig. 2A). It was also clear that Xyn8A produces longer chains at the shorter incubation time and these are then likely converted to smaller products with longer incubations. The Xyn5A and the Xyn10A end products did not show different patterns at 30 mins incubation compared with 14 hours of incubation, the only difference seen being more end products accumulating in the longer incubations. For the GH10/Ara43A, in both WAX and OSX, large amounts of xylotriose were seen in the shorter incubations (Supplementary Fig. 2A). We also examined the hydrolysis of the different xylans with the endoxylanase/accessory enzyme mixtures for only 30 mins (Supplementary Fig. 2B). Here also, there were no differences in the pattern of end products in all mixtures, other than the amounts of end products being lower than those observed for the 14 hour-incubations. Interestingly, the mixture of Xyn10B/Ara43A with the accessory enzymes on WAX at 30 mins incubation showed accumulation of xylobiose and xylotriose, which were not present in the same mixture incubated for 14 hours (Fig. 5 and Supplementary Fig. S2B).

## Discussion

The diverse endoxylanases in the genome of *B. intestinalis* alludes to the importance of xylans as nutrients to the life style of this bacterium, and the up-regulation of the diverse enzymes underscores the complexity of the xylans or nutrients that flow into the colon of the human host. The GH10 polypeptides with CBM insertions are widely distributed in the phylum Bacteroidetes, suggesting its importance in energy capture in this group of bacteria. It has been shown that the CBM insertions enhance the activity of BiXyn10A^14^, and this is likely a strategy used by the Bacteroidetes to enhance efficiency in energy capture as this type of enzyme is also found in xylanolytic *Prevotella* spp originating from the cow rumen. In the transcriptional analysis of two different *Bacteroides* spp. (*B. intestinalis* and *B. ovatus*) and in *Prevotella bryantii*, a member of the phylum Bacteroidetes from the cow rumen, grown on a xylan polysaccharide relative to a simple sugar, the most highly up-regulated gene in each case encoded a GH10 with CBM insertion^14^,^22^. Fascinatingly, aside from this unique enzyme, some xylan degrading *Bacteroides* spp. also have a potent bi-functional enzyme that is composed of a GH10 endoxylanase linked to a GH43 module. The GH43 catalytic module in this polypeptide is annotated as a β-xylosidase in the Genbank (Accession number: EDV05059.1), which based on bioinformatics is plausible since some members of this family function as β-xylosidases. However, based on our biochemical analysis, the GH43 in the GH10/GH43 polypeptide in *B. intestinalis* encodes an arabinofuranosidase. Thus, this embedded arabinofuranosidase cleaves the arabinose side chains that are commonly found to decorate the xylose backbone of different xylans, especially arabinoxylans. Removal of the side-chains by an arabinofuranosidase then allows accessibility and efficient hydrolysis of the β-1,4-linked xylose backbone. In fact, the importance of these enzymes are underscored by *B. intestinalis* having two homologs each of the GH10 with the CBM insertions and the GH10/Ara43 encoding genes on its genome. Furthermore, our observations strengthen the need for biochemical and functional studies of the gene products derived from the large sequence data or genes emanating from studies on the human microbiome.

It was surprising that the optimum temperatures of the xylan degrading enzymes encoded by the genes in the cluster analyzed in the present study are quite different from the body temperature of the host. However, the human colon, which is the natural habitat of the colonic Bacteroidetes, such as *B. intestinalis*, functions as a fermenter or bioreactor that experiences higher than normal body temperatures due to the fermentation process. Thus, the temperatures within which these enzymes function may not necessarily match the host normal body temperature, but rather a range of temperatures.

Our specific activity assays revealed that the five endoxylanases exhibited less hydrolytic activity on the insoluble substrate (OSX). Among the three xylan substrates, the best substrate for Xyn10B/Ara43A and Xyn8A was WAX, while Xyn10C, Xyn5A, and Xyn10A exhibited the highest activities on RAX. Besides the three xylans tested in the present study, there are several other human diets containing xylans, such as those derived from barley, rice, corn and sorghum. Therefore, the five endoxylanases should have different cleavage profiles and specific activities towards these substrates. The differences will facilitate efficient capture and utilization of nutrients from the different cereal sources to *B. intestinalis* in the human GIT, with subsequent nutritional and health impact on the human host. Recently, it was reported that *B. thetaiotaomicron* can simultaneously respond to multiple glycans and modify its metabolic responses (change the expression level of some glycan utilization genes) relative to glycan catabolism in dynamic glycan environments. Thus, similar to *B. thetaiotaomicron, B. intestinalis* and other Bacteroidetes likely exhibit a metabolic hierarchy dependent on the complexity of the glycan^27^,^28^. Analysis of the extensive xylan degradation system expressed by *B. ovatus*, which is highly similar to the XUS in *B. intestinalis*, has shown that *B. ovatus* harbors an extensive range of xylanolytic enzymes, capable of deconstructing different forms of xylan polysaccharide^29^. Thus, the strategy of these colonic xylanolytic Bacteroidetes may be highly conserved as proposed by Dodd and co-workers^19^.

The 14-hour incubation experiments were all end point assays, and therefore it was likely that the initial hydrolysis patterns ascribed to the endoxylanases were different, i.e., perhaps longer oligosaccharides were initially produced and further attacked by the enzymes to yield the type of end product patterns seen after the 14 hour-incubations. The shorter incubations of the enzymes with the substrates, in general, suggested that the hydrolytic patterns were not different between the shorter and longer incubations, with the only difference being that more end products accumulated at longer incubations. The synergistic activity of the Xyl43A and the Agu67A suggested that in the presence of complex xylans, such as WAX, RAX, and OSX, once the endoxylanases attack the xylan backbone to release oligosaccharides decorated with methyl-glucuronic acid, the Agu67A can cleave the side chains and thus expose the β-1,4 linked xylose chain for hydrolysis into xylose. Based on the modular organizations of the polypeptides, however, it is likely that the secreted enzymes, i.e., the endoxylanases and the Agu67A act on the xylan first, and their end products of undecorated xylooligosaccharides are transported into the cells. The Xyl43A, an intracellularly located enzyme, then acts on the xylooligosaccharides by cleaving them to xylose for fermentation through the pentose phosphate pathway.

When treated with the enzyme mixture consisting of the Xyn10B/Ara43A, the Xyl43A and the Agu67A, a large amount of arabinose and larger amounts of xylose were released compared to other enzyme mixtures in the present study. This observation indicated that the GH43 module confers arabinofuranosidase activity to the polypeptide, which enhanced the synergistic effect by cleaving the arabinofuranosyl side-chains from the xylose backbone, thus exposing the xylose-linked backbone to the endoxylanase. More arabinose released from WAX and RAX compared to OSX can be attributed to the higher arabinose content in arabinoxylan. In either the 30-min or 14-hours synergistic hydrolysis of xylan substrates, the predominant end products were monomers. This suggested that Xyl43A was efficient in the degradation of xylo-oligosaccharides and that the xylo-oligosaccharides were rapidly hydrolyzed after they were released. Therefore, very little to no xylo-oligosaccharides accumulated in the final hydrolytic products. As stated above, since a signal peptide is absent in Xyl43A, we predict that in the process of utilization of xylan substrates, the enzymes that initially attack the xylan substrates are secreted outside of the cytoplasm to degrade the complex xylans into small undecorated xylo-oligosaccharides. The oligosaccharides are then transported into the cytoplasm and further hydrolyzed by the Xy143A into xylose for metabolism and generation of energy and building blocks for the bacterium. Simultaneously, short chain fatty acids are produced by the bacterium as waste products but serve as nutrients for colonic epithelial cells^30^.

The combination of host factors, diet and gut microbial communities generate a complex environment that shape the niche of microorganisms within the community and for that matter the human gut microbiome. Human cereal diets, such as wheat, oat, and rye, provide a source of xylan for degradation by colonic microbial inhabitants. The vast arsenal of xylan targeting genes in the human colonic *Bacteroides* genus, including *B. intestinalis, B. ovatus, B. eggerthii*, and *B. cellulosilyticus*, points to an important niche within the human gut. This niche is related to a healthy diet rich in dietary fibers, especially xylans, a nutrient unavailable to the human host. Attack and degradation of these polysaccharides, as demonstrated here with cocktails of enzymes upregulated by *B. intestinalis* during growth on arabinoxylan, yield both monosaccharides and oligosaccharides that are easily transported into the *Bacteroides* cytoplasm and most likely also utilized by colonic microbiome members that practice cross-feeding, but lack the key depolymerization enzymes. Recent reports suggest that the human colonic microbiome harbors microorganisms capable of degrading cellulose through a cellulosomal machinery^31^,^32^. While the widespread distribution of microorganisms occupying the niche of energy capture from cellulose and its significance in the human colon still need investigation^33^, the results reported in the present study and others reported earlier^14^,^18^,^19^,^22^,^24^,^25^,^34^ point to an effective xylan/arabinoxylan degradation and utilization by members of the human colonic microbiome. Undoubtedly, the microbial degraders of recalcitrant polysaccharides constitute a vanguard that makes large amounts of energy available for capture and habitation in the human colon. Since the ability to capture energy is a primary requirement for colonization of an environment by a microbial community, our understanding of the interrelationships existing in the diverse members of the human colonic microbiome and the ability to manipulate them have been enhanced by the present report on xylan/arabinoxylan degradation by *B. intestinalis*. Furthermore, the capacity to degrade polysaccharides into monomeric sugars for fermentation by the human colonic bacteria not only impacts stability of the microbial community, but also makes fermentation end products, such as short chain fatty acids, available to the human gut colonocytes as an energy source.

## Materials and Methods

### Bacterial strains and materials

*B. intestinalis* DSM 17393^23^ was obtained from the Deutsche Sammlung von Mikrooganismen und Zellkulturen or German Collection of Microorganisms and cell Cultures (DSMZ, Braunschweig, Germany). The NEB Turbo chemical competent *Escherichia coli* cells and Q5 High-Fidelity 2X Master Mix (used for PCR) were purchased from New England Biolabs (Ipswich, MA). The *E. coli* BL21-CodonPlus (DE3)-RIL competent cells were obtained from Agilent (Santa Clara, CA). The pET-46 Ek/LIC vector was purchased from Novagen (San Diego, CA). The QIAprep Spin Miniprep kit was obtained from Qiagen, Inc. (Valencia, CA), and the Talon Metal Affinity resin was obtained from Clontech Laboratories, Inc. (Mountain View, CA). Amicon Ultra-15 centrifugal filter units with 10 kDa, 30 kDa, and 50 kDa molecular mass cutoffs (MMCOs) were obtained from Millipore (Billerica, MA). Soluble wheat arabinoxylan with medium viscosity (WAX), rye arabinoxylan (RAX) and xylo-oligosaccharides were obtained from Megazyme (Bray, Ireland), and oat spelt xylan (OSX) was purchased from Sigma-Aldrich (St. Louis, MO).

### Gene cloning, expression, and protein purification

The Rapid Annotation using Subsystem Technology (RAST) server (http://rast.nmpdr.org/)^35^ was used in analyzing the *B. intestinalis* genome sequence. Signal peptides and lipoprotein signal sequences were predicted using SignalP v4.1 (http://www.cbs.dtu.dk/services/SignalP/)^36^ and LipoP v1.0 (http://www.cbs.dtu.dk/services/LipoP/)^37^, respectively. Each of the genes encoding the five endoxylanases (BACINT_04197, BACINT_04202, BACINT_04210, BACINT_04213 and BACINT_04215) and two accessory enzymes (BACINT_04203 and BACINT_04205) was amplified from *B. intestinalis* DSM 17393 genomic DNA via PCR using NEB Q5 High-Fidelity 2X Master Mix. The PCR primers used for amplifying the genes are listed in Supplementary Table 1. The PCR products were purified and cloned into the pET-46 Ek/LIC vector according to the protocols described by the manufacturer (Stratagene). The ligated products were transformed into NEB Turbo competent *E. coli* cells, and the transformants were plated on lysogeny broth (LB) agar plates supplemented with 100 μg/ml ampicillin. The recombinant plasmids harboring the right inserts were confirmed by DNA sequencing (W. M. Keck Center for Comparative and Functional Genomics, University of Illinois). For gene expression, the recombinant plasmids containing the individual genes were introduced into *E. coli* BL21-CodonPlus(DE3) RIL competent cells by heat shock transformation and cultured overnight on LB agar plates supplemented with ampicillin (100 μg/ml) and chloramphenicol (50 μg/ml) at 37 °C. After 14 hours, individual colonies containing different genes were picked and inoculated into 10 ml fresh LB medium containing the same concentrations of ampicillin and chloramphenicol and incubated at 37 °C with vigorous shaking (250 rpm/min) for 6 hours. Each 10 ml culture was then transferred into a 1 liter LB medium in a 2.8 liter Fernbach flask and culturing continued at 37 °C until the optical density at 600 nm (OD_600_) reached 0.6. The gene expression inducer isopropyl β-D-thio-galactopyranoside (IPTG) was added to the culture at a final concentration of 0.1 mM to induce recombinant gene expression. The culturing was continued for 16 hours at a temperature of 16 °C with shaking at a speed of 200 rpm/min. The cells were harvested by centrifugation and resuspended in ice-cold lysis buffer (pH 7.9, 50 mM Tris-HCl, 300 mM NaCl) and ruptured by passage through an EmulsiFlex C-3 cell homogenizer (Avestin, Ottawa, Ontario, Canada). Each clarified supernatant, containing the cell contents, was obtained after centrifugation at 12,857× *g* for 30 mins at 4 °C and applied to a Talon cobalt resin already equilibrated with an equilibration buffer (pH 7.5, 50 mM Tris-HCl, 300 mM NaCl) according to the protocol of the manufacturer. The proteins bound to the cobalt-charged resin were eluted with an elution buffer (pH 7.5, 50 mM Tris-HCl, 300 mM NaCl, 250 mM imidazole). The collected fractions were pooled, concentrated into a final volume of 2 ml and centrifuged at 25,000× *g* for 5 mins to precipitate any denatured proteins. The recombinant proteins were further purified by size exclusion chromatography. For BiXyn10C, BiXyn10B/Ara43A and BiXyn10A, this step of purification involved an AKTAxpress fast protein liquid chromatography (FPLC) system equipped with a HiLoad 16/60 Superdex 200 column (GE Healthcare, Piscataway, NJ). The chromatography was developed with a buffer composed of 50 mM Tris-HCl, 150 mM NaCl, pH of 7.5. The proteins designated BiXyl43A, BiAgu67A, BiXyn8A and BiXyn5A were purified by the same method with the same buffer except for the column which was a HiLoad 16/60 Superdex 75 column. The protein concentrations of eluted fractions were determined by absorbance spectroscopy at 280 nm using a NanoDrop 1000 instrument (Thermo Scientific, Waltham, MA) with extinction coefficients of 168,485 M^−1^ cm^−1^, 180,570 M^−1^ cm^−1^, 117,940 M^−1^ cm^−1^, 137,420 M^−1^ cm^−1^, 144,535 M^−1^ cm^−1^, 78,395 M^−1^ cm^−1^, and 166,200 M^−1^ cm^−1^ for BiXyn10C, BiXyn10B/Ara43A, BiXyn8A, BiXyn5A, BiXyn10A, BiXyl43A, and BiAgu67A, respectively. Samples from eluted fractions were analyzed by 12% sodium dodecyl sulfate-polyacrylamide gel electrophoresis (SDS-PAGE) according to the method of Laemmli^38^, and protein bands were visualized by staining with Coomassie brilliant blue G-250. The highly purified protein fractions were used for the biochemical assays.

### Determination of optimal pH and temperature

The following buffers were used in the determination of the optimum pH of the *B. intestinalis* xylan-degrading enzymes: 50 mM sodium citrate, 150 mM NaCl (pH 4.0 to 6.0), 50 mM sodium phosphate, 150 mM NaCl (pH 6.0 to 7.5) and 50 mM Tris, 150 mM NaCl (pH 7.5 to 8.5). The five endoxylanases were incubated individually with 5 mg/ml WAX at different final enzyme concentrations (100 nM, 10 nM, 20 nM, 10 nM, 10 nM for BiXyn10C, BiXyn10B/Ara43A, BiXyn8A, BiXyn5A, and BiXyn10A, respectively) at 37 °C. The reducing sugars released were measured with the *para*-hydroxybenzoic acid hydrazide (pHBAH) method^39^ and a standard curve was derived using a series of concentrations of xylose. The optimum temperatures were determined by incubating each enzyme at the final concentrations mentioned above with 10 mg/ml WAX in the buffer in which optimal pH for activity was detected. The temperature range used was 25–60 °C, with an interval of 5 °C and the test for reducing ends, based on the pHBAH method, was the assay used for enzymatic activity. For BiXyl43A, the optimal pH and temperature were determined by incubating the purified enzyme (a final concentration of 100 nM) with xylobiose (1 mg/ml) in the series of buffer mentioned above at 37 °C for 10 mins in a total volume of 50 μl. Then the mixture was heated at 100 °C for 10 min. The increase in the amount of reducing sugar was determined by pHBAH assay. Similarly, the optimal temperature was determined in the optimal pH at a temperature ranging from 15 to 60 °C with an interval of 5 °C.

### Specific activities of multiple endoxylanases on xylan substrates

To investigate the enzymatic activities of the five xylanases on polysaccharide substrates (WAX, RAX and OSX), the specific activity for each enzyme was determined individually at the optimal pH and temperature, with the final enzyme concentration indicated in brackets: BiXyn10C (100 nM); BiXyn10B/Ara43A (10 nM); BiXyn8A (10 nM); BiXyn5A (20 nM); and BiXyn10A (100 nM), respectively. Fifty microliter aliquots were removed at regular time intervals (3, 6 and 9 mins) from the reaction mixtures, and the concentrations of reducing ends released with time were assayed using the pHBAH method with known xylose concentrations used in plotting a standard curve.

### Hydrolysis of xylo-oligosaccharides by BiXyl43A

The hydrolytic activity of BiXyl43A against xylo-oligosaccharides (xylobiose, X2; xylotriose, X3; xylotetraose, X4, xylopentaose; X5, and xylohexaose, X6) was assayed by incubating each substrate (2.5 mg/ml, final concentration) with BiXyl43A (0.25 μM, final concentration) in a phosphate buffer (pH 6.5, 50 mM sodium phosphate, 150 mM NaCl) at 37 °C for 15 mins in a total volume of 50 μl. The enzyme was heat inactivated after the reaction, and 20 μl of each sample was diluted with distilled water to 400 μl and the end products of hydrolysis were analyzed by high-performance anion-exchange chromatography coupled with pulse amperometric detection (HPAEC-PAD) as described in our previous report^40^.

### Hydrolysis of alduronic acids

The enzyme BiXyl43A or BiAgu67A (0.5 μM, final concentration) was incubated with alduronic acids (1.2 mg/ml, final concentration) at 37 °C individually or together to investigate their potential for synergistic activity. The enzyme was inactivated by heating at 100 °C for 10 mins after 30 mins reaction. The reaction end products were further analyzed by HPAEC-PAD as described above. Xylose and xylo-oligosaccharides (X2 to X4) were analyzed as standards. Calibration curves of a series of concentrations of the monosaccharide and oligosaccharides were generated and the concentrations in the reaction mixtures were calculated.

### Hydrolysis of WAX, RAX and OSX

To analyze the hydrolytic end products of the five endoxylanases on xylan polysaccharide substrates (WAX, RAX and OSX), the enzymes (final concentration of 0.5 μM) were incubated with each substrate [0.5% (wt/v), final concentration] in citrate buffer (50 mM sodium citrate, 150 mM NaCl, pH 6.0) at 37 °C for 30 mins and 14 hours. The synergistic activities of the xylanases with the two putative accessory enzymes (BiXyl43A and BiAgu67A) were also investigated. The accessory enzymes were incubated with each endoxylanase at 37 °C individually or together. The final concentration of each enzyme was equivalent (0.5 μM). After 30 mins and 14 hours of incubation, the hydrolytic reactions were terminated by heat treatment at 100 °C for 10 mins. The reducing sugar concentrations were determined using the pHBAH assay with xylose as the standard. The concentrations of the end products of hydrolysis were quantitatively analyzed by HPAEC-PAD, as described in our previous report^40^. Arabinose, xylose, and xylo-oligosaccharides (X2 to X6) were used as standards. The calibration curves were derived with known concentrations of arabinose, xylose, and xylo-oligosaccharides (X2 to X6).

## Additional Information

**How to cite this article**: Wang, K. *et al. Bacteroides intestinalis* DSM 17393, a member of the human colonic microbiome, upregulates multiple endoxylanases during growth on xylan. *Sci. Rep.* **6**, 34360; doi: 10.1038/srep34360 (2016).

## Supplementary Material

Supplementary Information

## Figures and Tables

**Table 1:** pH and temperature optima of xylanolytic enzymes in PUL.

Enzyme	pH optima	Temperature optima
BiXyn10C	7.0	45 °C
BiXyn10B/Ara43A	5.5	50 °C
BiXyn8A	6.0	50 °C
BiXyn5A	6.0	50 °C
BiXyn10A	6.0	45 °C
BiXyl43A	6.5	35 °C

**Table 2:** Specific activities of the five endoxylanases with WAX, RAX and OSX as substrates.

Substrate	Specific activities (μmol/sec/μmol)				
	BiXyn10C	BiXyn10B/Ara43A	BiXyn8A	BiXyn5A	BiXyn10A
WAX	37.6 ± 4.3	1682.6 ± 56.3	1547.7 ± 2.6	254.3 ± 5.1	250.1 ± 4.2
RAX	39.9 ± 0.7	1634.6 ± 158.3	1227.7 ± 150.5	554.7 ± 30.3	351.1 ± 17.8
OSX	23.2 ± 1.6	619.8 ± 31.1	743.9 ± 21.8	43.6 ± 2.7	188.0 ± 2.0
