# Supplementary material for: Bacteroides intestinalis DSM 17393, a member of the human colonic microbiome, upregulates multiple endoxylanases during growth on xylan
Source: Sci Rep. 2016 Sep 29;6:34360. doi: 10.1038/srep34360 (PMC5041131; doi:10.1038/srep34360)

Supplemental materials

Bacteroides intestinalis DSM 17393, a member of the human colonic microbiome, upregulates multiple endoxylanases during growth on xylan

Kui Wang[1,2], Gabriel V. Pereira[1,2,3], Janaina J. V. Cavalcante[1,2], Meiling Zhang[1,2,3], Roderick Mackie[1,2,3] and Isaac Cann[1,2,3,4,*]

Energy Biosciences Institute[1], Carl R. Woese Institute for Genomic Biology[2], Department of Animal Science[3], Department of Microbiology[4], University of Illinois at Urbana-Champaign, Urbana, IL 61801, USA

Supplemental Table 1. Primer sequences used in this study.

Gene name	Direction	Nucleotide sequence (5'→3')[a]
BiXyn10C	Forward	GACGACGACAAGTGCGAGGATAATAAGATGGAATGGGGTAC
	Reverse	GAGGAGAAGCCCGGTTATTGATCAGCGGGAGTTTCTTCCTC
BiXyn10B/Ara43A	Forward	GACGACGACAAGGCAGACCCGACTCTAAAAGATATTCTC
	Reverse	GAGGAGAAGCCCGGTTAATTCGTATAGTTTCCTGTGGCAT
BiXyn8A	Forward	GACGACGACAAGCATCCTGTTCAGGAAGACAGTAGTGGG
	Reverse	GAGGAGAAGCCCGGCTACTTGATAATCCGGAAATTGCC
BiXyn5A	Forward	GACGACGACAAGGACGAGACTATTCCTGAGCAGCCG
	Reverse	GAGGAGAAGCCCGGTTAATTTGCTAAGGAGACATTAGCTAT
BiXyn10A	Forward	GACGACGACAAGGTGGACGACAAACCGTTGGCATTCGAAG
	Reverse	GAGGAGAAGCCCGGTTATTTTCCAGCCAATCCATCAGCAAAAC
BiXyl43A	Forward	GACGACGACAAGATGAAGAAAGAAAAAAGATACTTGGTACCCGGAGAT
	Reverse	GAGGAGAAGCCCGGTTAATCCAGTCCTTCAATGGTGACGATCT
BiAgu67A	Forward	GACGACGACAAG GAAGACGGCAGCCGCTTG
	Reverse	GAGGAGAAGCCCGGTTATCTTCTTTCATTCAGCATTTTG

[a] Sequences underlined denote the incorporated T4 exonuclease digestion sites from pET-46b Ek-LIC vector.

Fig. S1 Biochemical characterization of xylan utilization loci. Enzymatic activity reported relative to optimal pH (A) and temperature (B) conditions. Error bars represent standard deviation for three replicates

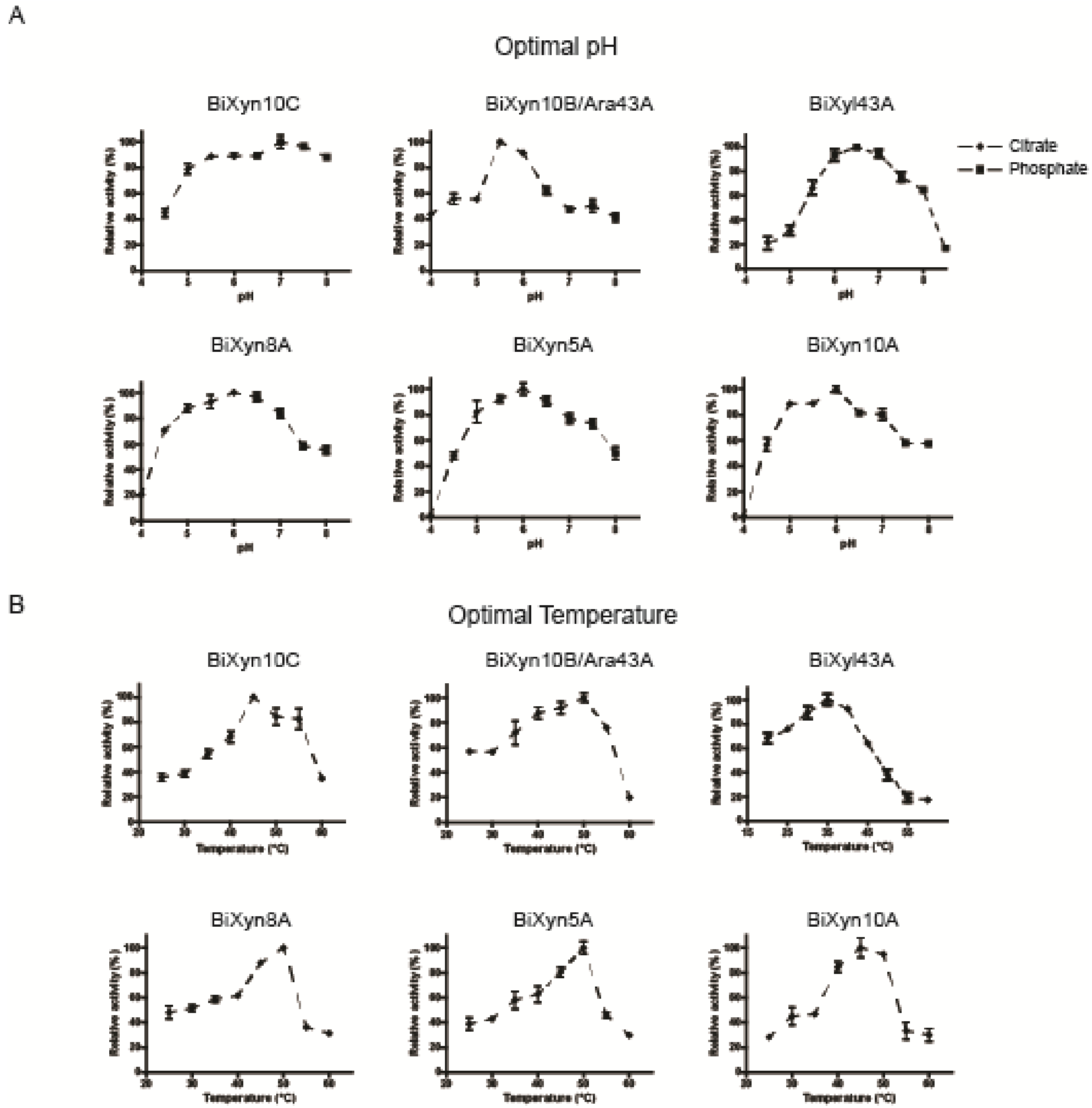

Fig. S2 30-min hydrolysis of xylan substrates. The final concentration for each enzyme was 0.5 μM, the final hydrolysis products were identified and quantified by HPLC, Arabinose (A1), xylose (X1) and xylo-oligosaccharides (X2 to X6) were mixed and analyzed by HPLC to serve as standards for the assignment of the released products. Calibration curves were constructed with known concentrations of A1 and X1-X6. The concentrations of each sugar were calculated by fitting the peak area into calibration curve.

(A) 30-min hydrolysis of xylan substrates (WAX, RAX and OSX) by the five putative endoxylanases.

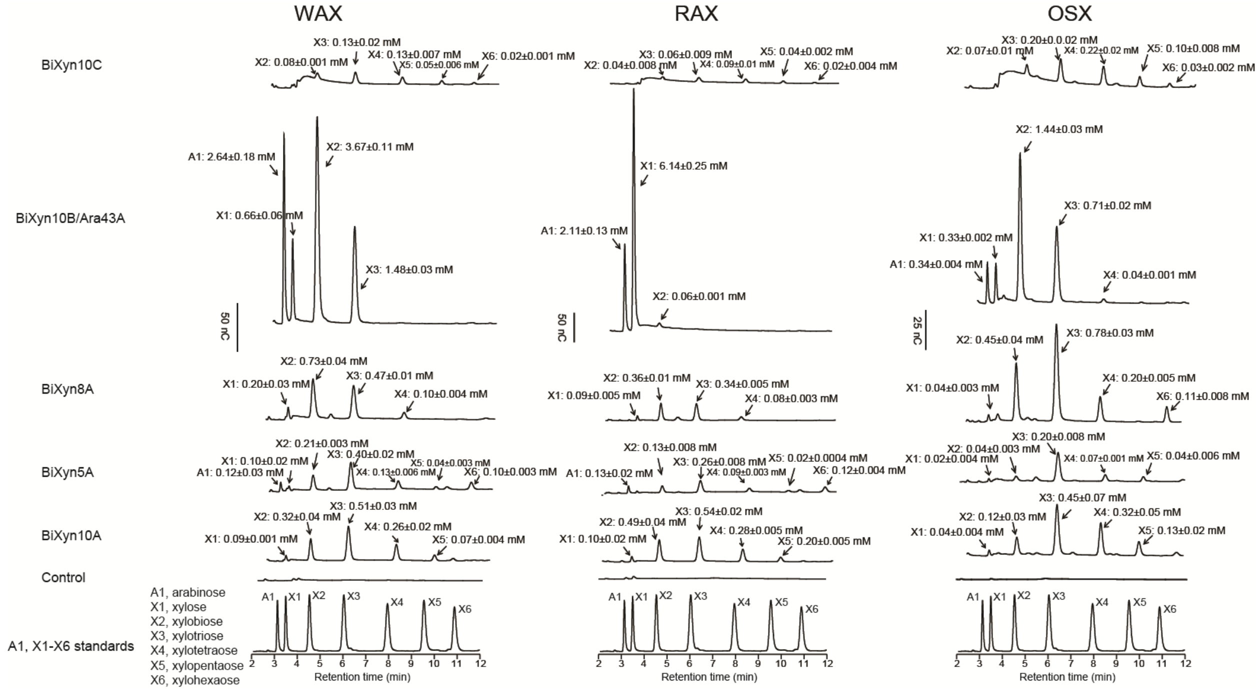

Fig. S2 (B) 30-min synergistic hydrolysis of xylan substrates of multiple endoxylanases with BiXyl43A and BiAgu67A towards WAX, RAX and OSX.

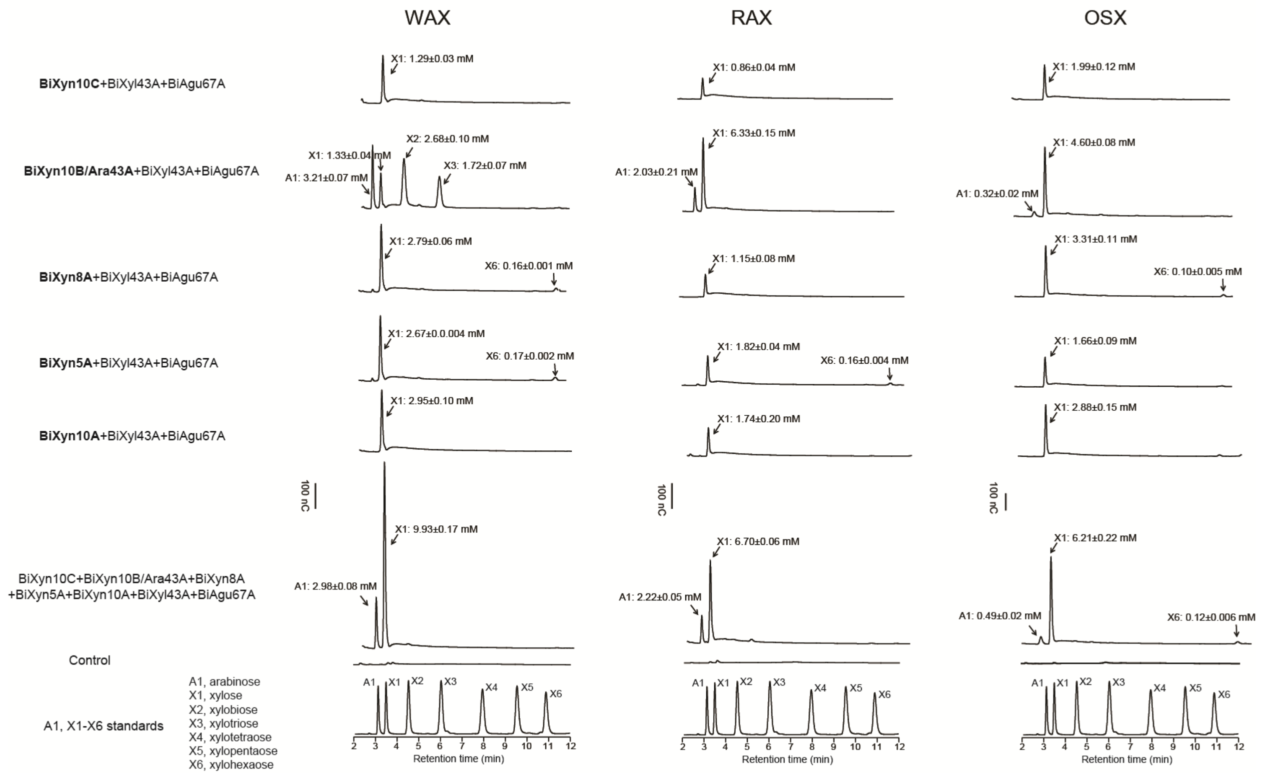

Supplement: Supplementary Information [file srep34360-s1.pdf]
